# Three 4-(4-fluoro­phen­yl)piperazin-1-ium salts containing organic anions: supra­molecular assembly in one, two and three dimensions

**DOI:** 10.1107/S2056989020006398

**Published:** 2020-05-15

**Authors:** Chayanna Harish Chinthal, Hemmige S. Yathirajan, Sreeramapura D. Archana, Sabine Foro, Christopher Glidewell

**Affiliations:** a Department of Studies in Chemistry, University of Mysore, Manasagangotri, Mysuru-570 006, India; b Institute of Materials Science, Darmstadt University of Technology, Alarich-Weiss-Strasse 2, D-64287 Darmstadt, Germany; c School of Chemistry, University of St Andrews, St Andrews, Fife KY16 9ST, UK

**Keywords:** piperazines, piperazinium salts, crystal structure, mol­ecular conformation, hydrogen bonding, supra­molecular assembly

## Abstract

Three salts containing the 4-(4-fluoro­phen­yl)piperazin-1-ium cation have been prepared and structurally characterized. In 4-(4-fluoro­phen­yl)piperazin-1-ium 2-hy­droxy-3,5-di­nitro­benzoate, C_10_H_14_FN_2_^+^·C_7_H_3_N_2_O_7_^−^, (I), the anion contains an intra­molecular O—H⋯O hydrogen bond, and it has a structure similar to that of the picrate ion. The cations and anions are linked into [001] chains of rings by a combination of two three-centre N—H⋯(O)_2_ hydrogen bonds. The anion in 4-(4-fluoro­phen­yl)piperazin-1-ium hydrogen oxalate, C_10_H_14_FN_2_^+^·C_2_HO_4_^−^, (II), is planar, and the cations and anions are linked into (100) sheets by multiple hydrogen bonds including two-centre N—H⋯O, three-centre N—H⋯(O)_2_, O—H⋯O, C—H⋯O and C—H⋯π(arene) types. In 4-(4-fluoro­phen­yl)piperazin-1-ium hydrogen (2*R*,3*R*)-tartrate monohydrate, C_10_H_14_FN_2_^+^·C_4_H_5_O_6_^−^·H_2_O, (III), the anion exhibits an approximate non-crystallographic twofold rotation symmetry with anti­periplanar carboxyl groups. A combination of eight hydrogen bonds, encompassing two- and three-centre N—H⋯O systems, O—H⋯O and C—H⋯π(arene) types, link the independent components into a three-dimensional framework. Comparisons are made with some related structures.

## Abstract 2

Three salts containing the 4-(4-fluoro­phen­yl)piperazin-1-ium cation have been prepared and structurally characterized.

## Chemical context   

*N*-(4-fluoro­phen­yl)piperazine (4-FPP) is a major metabolite (Keane *et al.*, 1982 ▸; Sanjuan *et al.*, 1983 ▸) of the sedative and hypnotic drug niaprazine (*N*-{4-[4-(4-fluoro­phen­yl)piperazin-1-yl]butan-2-yl}pyridine-3-carboxamide), used in the treatment of autistic disorders (Rossi *et al.*, 1999 ▸). 4-FPP itself has mildly psychedelic and euphorigenic properties and, in this respect, it exhibits effects similar to those of the related compound *N*-(4-meth­oxy­phen­yl)piperazine (MeOPP), also used as a recreational drug (Nagai *et al.*, 2007 ▸).

We have recently reported the structure of MeOPP and those of a number of salts derived from it (Kiran Kumar *et al.*, 2019 ▸, 2020 ▸). With the similarities of action between MeOPP and 4-FPP in mind, we have now prepared and structurally characterized a selection of salts derived from 4-FPP, namely 4-(4-fluoro­phen­yl)piperazin-1-ium 2-hy­droxy-3,5-di­nitro­benzoate (I), 4-(4-fluoro­phen­yl)piperazin-1-ium hydrogenoxalate (II) and 4-(4-fluoro­phen­yl)piperazin-1-ium (2*R*,3*R*)-hydrogentartrate, which crystallizes from ethyl acetate as a monohydrate (III).
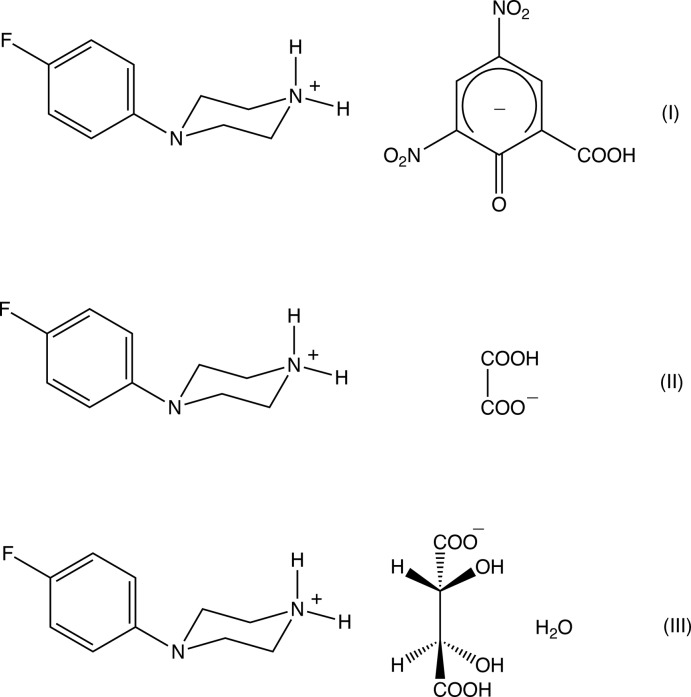


## Structural commentary   

**Figure 1:**
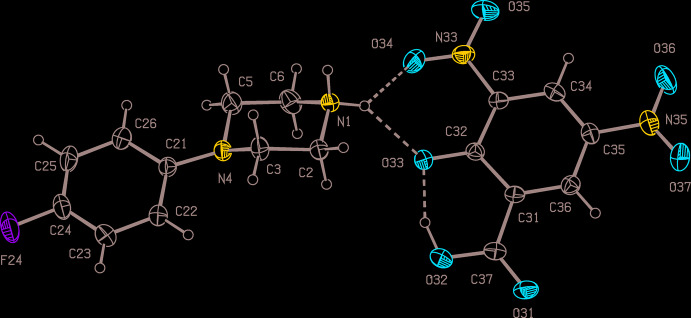
The independent components of compound (I) showing the atom-labelling scheme and the hydrogen bonds (drawn as dashed lines) within the asymmetric unit. Displacement ellipsoids are drawn at the 30% probability level.

Compounds (I)–(III) are all 1:1 salts (Figs. 1 ▸–3 ▸ ▸) in which a single proton has been transferred from the diprotic acid component to the 4-(4-fluoro­phen­yl)piperazine component: of these, (I) and (II) both crystallize in solvent-free form, but (III) crystallizes as a monohydrate. Since a single enanti­omer of tartaric acid, the (2*R*,3*R*) form, was used in the synthesis of (III), which occurred under very mild conditions unlikely to induce any stereochemical changes, only a single enanti­omer is present in the product, which therefore crystallizes in a Sohncke space group containing neither inversion nor reflection (mirror or glide) operations, here *P*2_1_2_1_2_1_.

In each of (I)–(III), the piperazine ring adopts an almost perfect chair conformation, with the 4-fluoro­phenyl substit­uent occupying an equatorial site. The value of the ring-puckering angle θ (Cremer & Pople, 1975 ▸), calculated for the atom sequence (N1,C2,C3,N4,C5,C6), ranges from to 2.0 (4)° in (III) to 4.85 (12)° in (II), very close to the ideal value of zero for a perfect chair form (Boeyens, 1978 ▸).

In the anions in each of compounds (I)–(III), the location of the remaining acidic H atom was initially deduced from difference-Fourier maps, and then confirmed by refinement of the atomic coordinates, reinforced by inspection of the final difference-Fourier map and of the relevant C—O bond lengths, where the single and double bonds have distances entirely typical of their types (Allen *et al.*, 1987 ▸).

In the anion of compound (I) (Fig. 1 ▸), it is the phenolic proton that has been transferred rather than the carboxyl proton; this was confirmed as described above. The other bond lengths in this anion show some inter­esting features. Firstly, the distance C32—O33, 1.2719 (18) Å, is much closer to the values typically found in cyclo­hexa­nones (mean value, 1.211 Å) than to those found in phenols (mean value 1.362 Å); secondly, the bond lengths C31—C32 and C32—C33, 1.441 (2) and 1.4318 (19) Å, respectively, are much longer than the other C—C distances in this ring, which lie in the range from 1.368 (2) to 1.388 (2) Å. The bond lengths in the anion, taken together, thus indicate extensive delocalization of the negative charge away from atom O33 and onto the aromatic ring atoms C31,C33,C34,C35,C36 (*cf*. Scheme), as has been observed in picrate (2,4,6-tri­nitro­phenolate) anions (Sagar *et al.*, 2017 ▸; Shaibah *et al.*, 2017*a* ▸,*b* ▸). However, this anion is not completely planar: the substituents at atoms C31, C33 and C35 make dihedral angles with the plane of the ring of 7.62 (16), 9.31 (12), and 10.9 (2)°, respectively.

**Figure 2:**
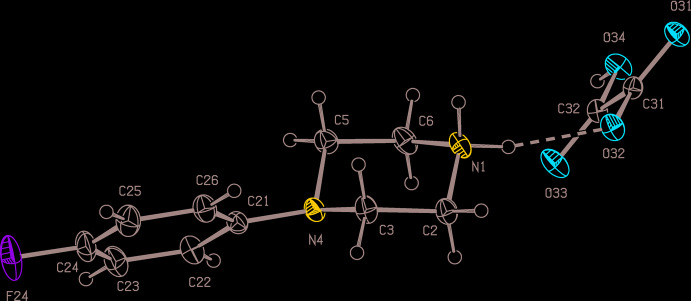
The independent components of compound (II) showing the atom-labelling scheme and the hydrogen bonds (drawn as dashed lines) within the asymmetric unit. Displacement ellipsoids are drawn at the 30% probability level.

By contrast, the anion in compound (II) (Fig. 2 ▸) is planar: the r.m.s. deviation from the mean plane through the non-H atoms is only 0.014 Å, with a maximum individual deviation from this plane of 0.0186 (6) Å for atom O34. In the anion of (III), the carboxyl and carboxyl­ate groups are anti­periplanar, as shown by the value of −178.81 (10)° for the torsional angle C31—C32—C33—C34, while the disposition of the two hydroxyl groups is indicated by the value of −66.5 (3)° for the torsional angle O33—C32—C33—O34. Together with the torsional angles O31—C32—C33—C34 and O36—C34—C33—C32, 64.7 (4)° and 59.5 (3)°, respectively, the torsional angles overall indicate that the non-H atoms in this anion exhibit approximate, although non-crystallographic, two-fold rotation symmetry.

## Supra­molecular features   

Within the selected asymmetric unit for compound (I) (Fig. 1 ▸), the anion contains an intra­molecular O—H⋯O hydrogen bond (Table 1 ▸), generating an *S*(6) motif (Etter, 1990 ▸; Etter *et al.*, 1990 ▸; Bernstein *et al.*, 1995 ▸), and the cation and anion are linked by a three-centre N—H⋯(O)_2_ system to form an 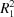
(6) motif. Ion pairs of this type, which are related by the *c* glide plane at *y* = 0.25, are linked by a second, rather asymmetric, three-centre system *via* an 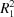
(4) motif to form a chain of rings running parallel to [001] (Fig. 4 ▸). There is also a short C—H⋯O contact (Table 1 ▸), which lies within the chain of rings: however, the small C—H⋯O angle indicates that the inter­action energy is likely to be very small (Wood *et al.*, 2009 ▸), so that this is probably best regarded as an adventitious contact of little structural significance.

The component ions in compound (II) (Fig. 2 ▸) are linked by a single N—H⋯O hydrogen bond (Table 2 ▸). The ion pairs, which are related by a 2_1_ screw axis along (0.5, *y*, 0.25), are linked by a combination of an asymmetric three-centre N—H⋯(O)_2_ hydrogen bond and a two-centre O—H⋯O hydrogen bond (Table 2 ▸) to form a complex chain of rings running parallel to the [010] direction (Fig. 5 ▸). This chain is reinforced by two C—H⋯O hydrogen bonds, involving methyl­ene atoms C2 and C6 as the donors. However, the combination of the C—H⋯O hydrogen bond having methyl­ene atom C5 as the donor and the C—H⋯π(arene) hydrogen bond having atom C2 as the donor links ion pairs, which are related by the *c* glide plane at *y* = 0.75, to form a second chain of rings, this time running parallel to the [001] direction (Fig. 6 ▸). The combination of chains along [010] and [001] generates a complex sheet lying parallel to (100). There is a fairly short O⋯C contact between inversion-related anions, with a distance O31⋯C32^i^ [symmetry code: (i) 1 − *x*, 1 − *y*, 2 − *z*] of 3.0108 (14) Å, but it is unclear whether this has any structural significance.

**Figure 3:**
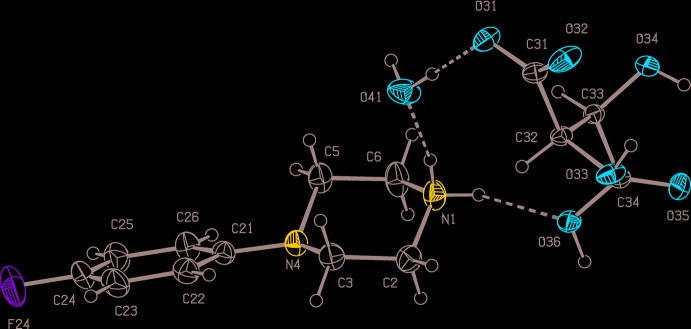
The independent components of compound (III) showing the atom-labelling scheme and the hydrogen bonds (drawn as dashed lines) within the asymmetric unit. Displacement ellipsoids are drawn at the 30% probability level.

**Figure 4:**
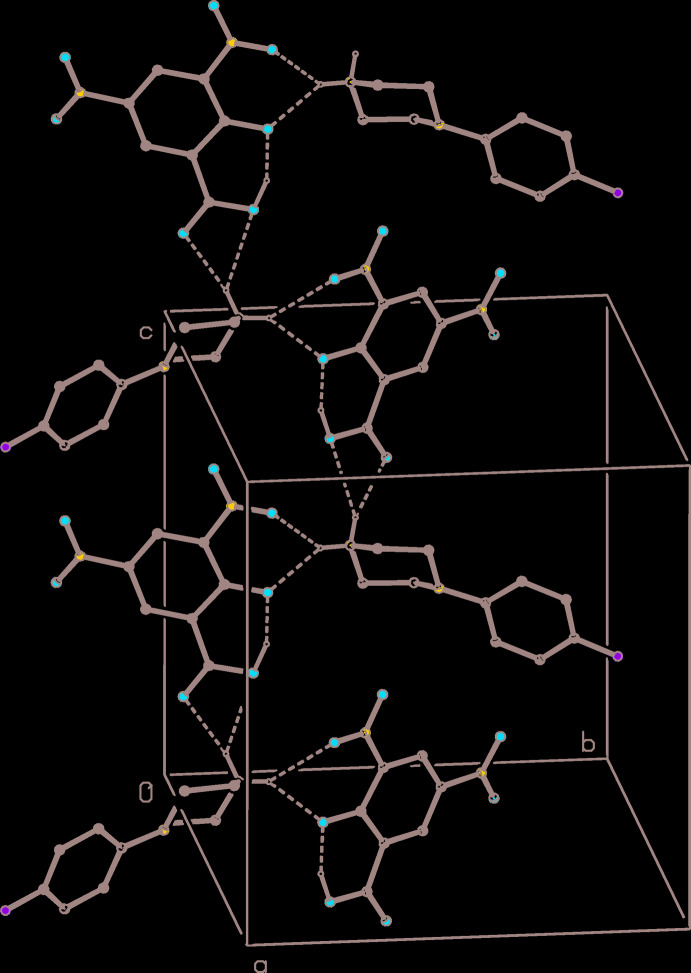
Part of the crystal structure of compound (I) showing the formation of a hydrogen-bonded chain of rings running parallel to [001]. Hydrogen bonds are drawn as dashed lines and, for the sake of clarity, the H atoms bonded to C atoms have been omitted.

**Figure 5:**
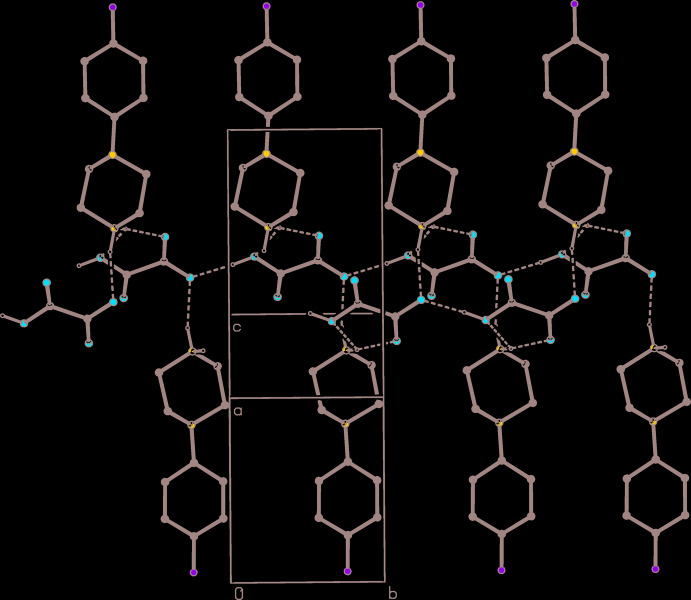
Part of the crystal structure of compound (II) showing the formation of a hydrogen-bonded chain of rings running parallel to the [010] direction. Hydrogen bonds are drawn as dashed lines and, for the sake of clarity, the H atoms bonded to C atoms have been omitted.

**Figure 6:**
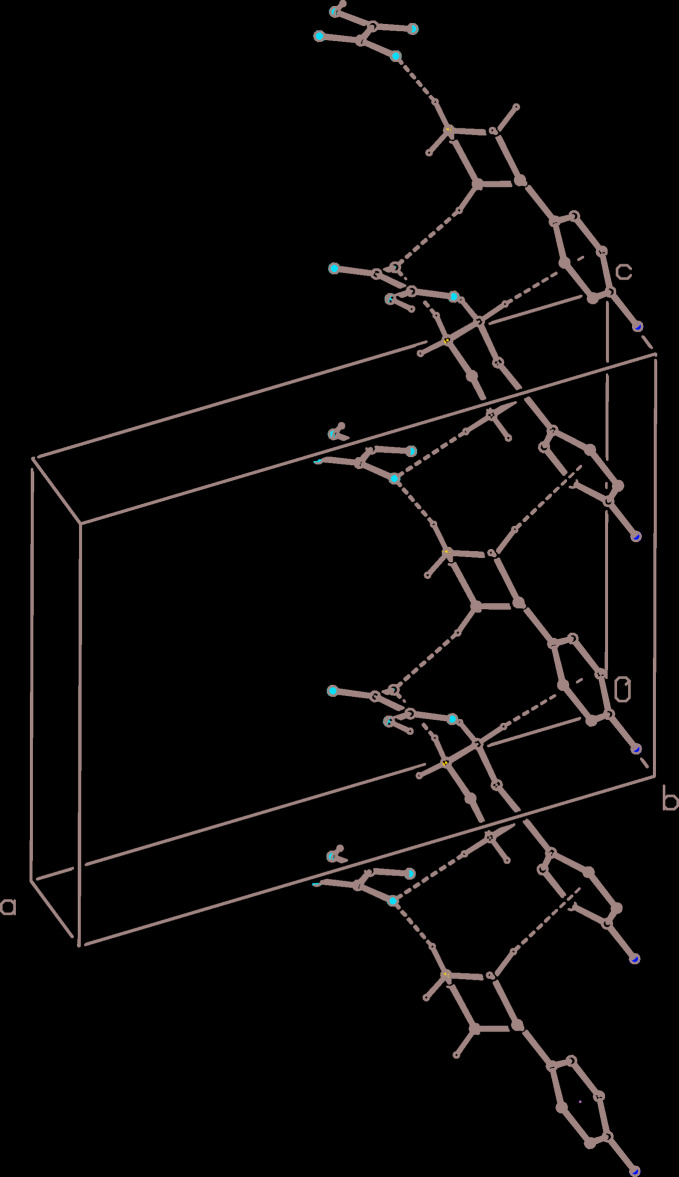
Part of the crystal structure of compound (II) showing the formation of a chain of rings running parallel to the [001] direction and built from C—H⋯O and C—H⋯π(arene) hydrogen bonds. Hydrogen bonds are drawn as dashed lines and, for the sake of clarity, the H atoms bonded to the C atoms not involved in the motif shown have been omitted.

**Figure 7:**
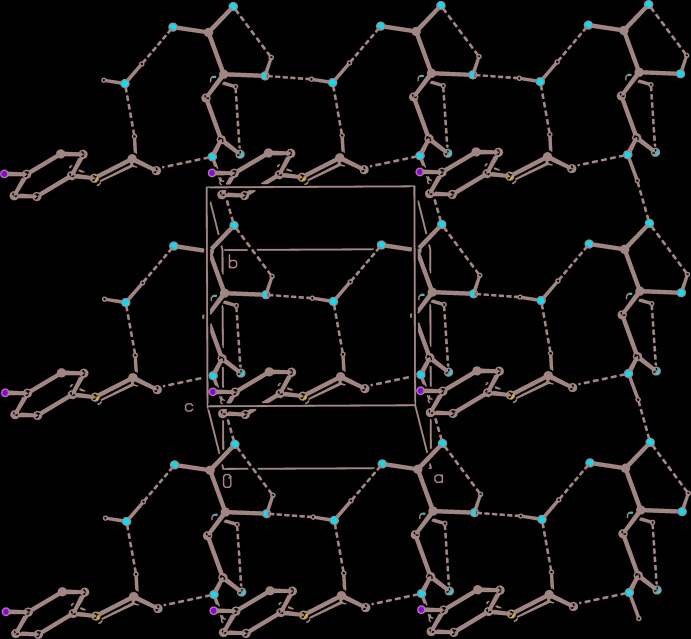
Part of the crystal structure of compound (III) showing the formation of a hydrogen-bonded sheet lying parallel to (001). Hydrogen bonds are drawn as dashed lines and, for the sake of clarity, the H atoms bonded to C atoms have been omitted.

**Figure 8:**
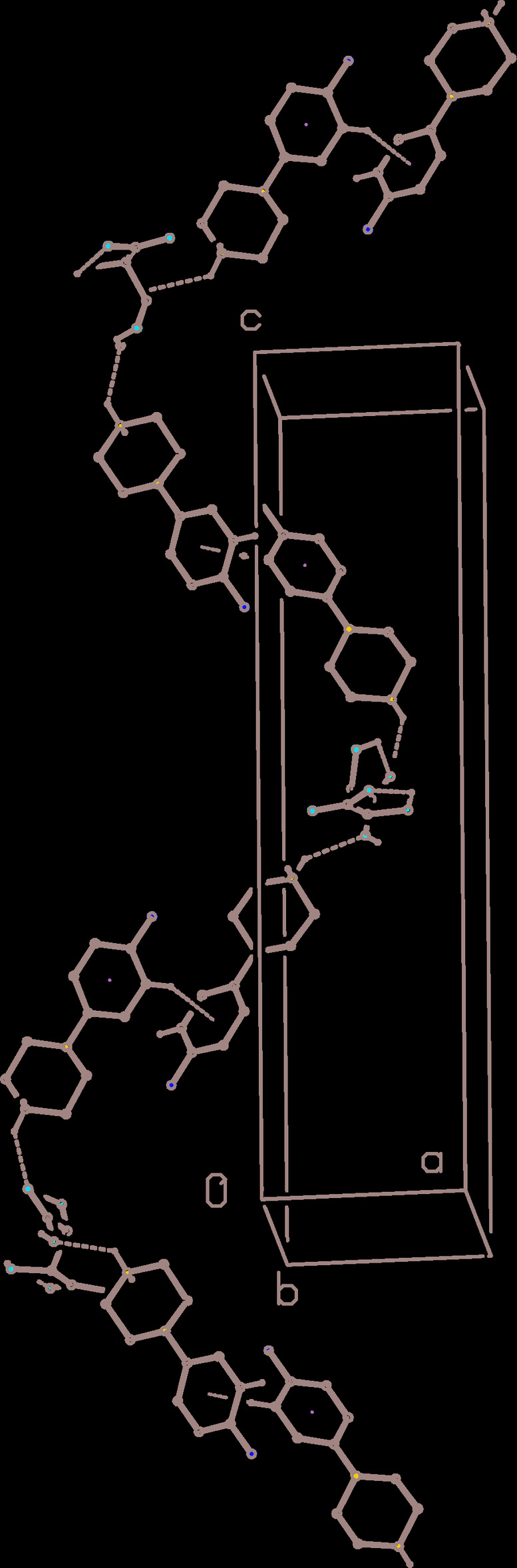
Part of the crystal structure of compound (III) showing the formation of a hydrogen-bonded chain of cations and anions running parallel to the [001] direction. Hydrogen bonds are drawn as dashed lines and, for the sake of clarity, the water mol­ecules and the H atoms not involved in the motif shown have been omitted.

The supra­molecular assembly in the monohydrate (III) is more complex than that in either (I) or (II), and it is three-dimensional as opposed to the one- and two-dimensional assembly in (I) and (II), respectively. However, the three-dimensional assembly in (III) can readily be analysed in terms of some simpler sub-structures (Ferguson *et al.*, 1998*a* ▸,*b* ▸; Gregson *et al.*, 2000 ▸). Within the asymmetric unit (Fig. 3 ▸), the components are linked by two N—H⋯O hydrogen bonds and one O—H⋯O hydrogen bond (Table 3 ▸), forming a compact aggregate containing an 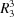
(11) motif (Fig. 3 ▸). The inter-aggregate hydrogen bonds having atoms O36 and O41 as the donors link aggregates related by translation to form a sheet lying parallel to (001) in the domain 0.5 < *z* < 1.0 (Fig. 7 ▸). A second sheet of this type, related to the first by the 2_1_ screw axes parallel to [100], lies in the domain 0 < *z* < 0.5 and adjacent sheets of this type are linked into a bilayer by a combination of N—H⋯O and O—H⋯O hydrogen bonds (Table 3 ▸). Finally, the bilayers are linked into a continuous three-dimensional structure by a single C—H⋯π(arene) hydrogen bond: in combination with the N—H⋯O hydrogen bond linking the ion pairs within the asymmetric unit, this C—H⋯π inter­action generates a chain running parallel to the [001] direction (Fig. 8 ▸), thereby linking adjacent bilayers.

## Related structures   

It is of inter­est briefly to compare the structures reported here with those of some closely related compounds. An obvious comparison is between compound (I), reported here and the analogous salt (IV) derived from MeOPP (Kiran Kumar *et al.*, 2019 ▸). Although (I) and (IV) both crystallize in space-group type *P*2_1_/*c*, their unit-cell dimensions are very different, as is the manner of their supra­molecular assembly. Thus, in the structure of (IV), a combination of N—H⋯O and C—H⋯O hydrogen bonds links the component ions into a chain of centrosymmetric rings in which rings of 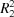
(10) and 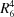
(16) types alternate, with chains of this type linked by C—H⋯π(arene) hydrogen bonds to form a three-dimensional network, as compared with the one-dimensional assembly in (I). Thus a change in one small passive substituent between compounds (I) and (IV) is associated with a considerable change in the crystal structure. The constitution of compound (II) has some resemblance to the hydrogensuccinate (V) and hydrogenfumarate (VI) salts of MeOPP, in both of which anions exhibits some disorder (Kiran Kumar *et al.*, 2019 ▸). In each of (V) and (VI) the component ions are linked by a combination of O—H⋯O and N—H⋯O hydrogen bonds to form sheets, which are in turn linked into a three-dimensional assembly by C—H⋯π(arene) hydrogen bonds, as compared to the two dimensional assembly in (II). We also note that structures have been reported for 4-[bis­(4-fluoro­phen­yl)meth­yl)piperazine (VII) (Dayananda *et al.*, 2012*a* ▸), and for its 1-acetyl derivative (VIII) (Dayananda *et al.*, 2012*b* ▸), both of which are inter­mediates on the synthetic pathway to the calcium-channel blocker flunarizine, 1-[bis­(4-fluoro­phen­yl)meth­yl]-4-cinnamyl-piperazine (IX) (Prasanna & Row, 2001 ▸).
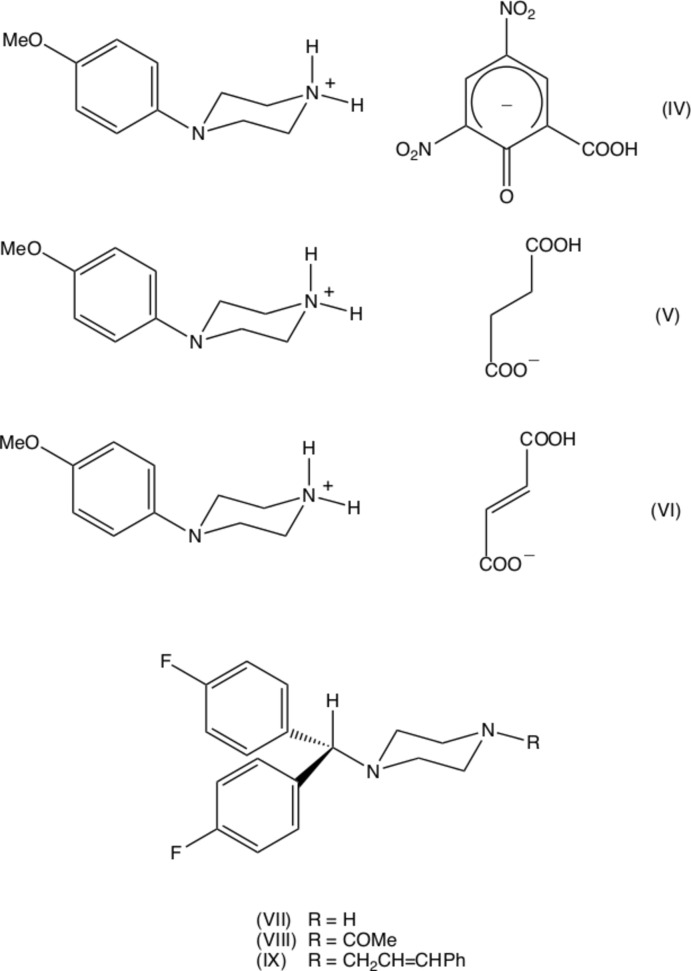


## Synthesis and crystallization   

All starting materials were obtained commercially, and all were used as received. For the preparation of compounds (I)–(III), *N*-(4-fluoro­phen­yl)piperazine (100 mg, 0.55 mmol) was dissolved in methanol (10 ml) and a solution of the appropriate acid (0.55 mmol) in methanol (10 ml) [2-hy­droxy-3,5-di­nitro­benzoic acid, 125.5 mg for (I), oxalic acid, 49.5 mg for (II), and (2*R*,3*R*)-tartaric acid, 82.5 mg for (III)] was then added; the mixtures were briefly stirred at 323 K before being set aside at ambient temperature to crystallize. After two days, the resulting solid products were collected by filtration and dried in air. Crystals suitable for single-crystal X-ray diffraction were grown by slow evaporation, at ambient temperature and in the presence of air, of solutions in ethyl acetate for (I) and (III), or in methanol for (II): m.p. (I) 460–463 K, (II) 421–425 K, (III) 437–441 K.

## Refinement   

Crystal data, data collection and refinement details are summarized in Table 4 ▸. All H atoms were located in difference-Fourier maps. The H atoms bonded to C atoms were then treated as riding atoms in geometrically idealized positions with C—H distances 0.93 Å (aromatic), 0.97 Å (CH_2_), or 0.98 Å (aliphatic C—H) and with *U*_iso_(H) = 1.2*U*_eq_(C). The H atoms bonded to N or O atoms were refined with *U*_iso_(H) = 1.2*U*_eq_(N) or 1.5*U*_eq_(O), giving the N—H and O—H distances shown in Tables 1 ▸–3 ▸ ▸. In the absence of significant resonant scattering in compound (III), the Flack *x* parameter (Flack, 1983 ▸) was indeterminate (Flack & Bernardinelli, 2000 ▸): thus the value of *x*, calculated (Parsons *et al.*, 2013 ▸) using 683 quotients of type [(*I* ^+^) − (*I* ^−^)]/[(*I* ^+^) + (*I* ^−^)], was −1.5 (7). Since a single enanti­omer, the (2*R*,3*R*) form, of tartaric acid was used in the preparation of compound (III), the absolute configuration in the crystal of (III) was set on this basis.

## Supplementary Material

Crystal structure: contains datablock(s) global, I, II, III. DOI: 10.1107/S2056989020006398/wm5557sup1.cif

Structure factors: contains datablock(s) I. DOI: 10.1107/S2056989020006398/wm5557Isup2.hkl

Structure factors: contains datablock(s) II. DOI: 10.1107/S2056989020006398/wm5557IIsup3.hkl

Structure factors: contains datablock(s) III. DOI: 10.1107/S2056989020006398/wm5557IIIsup4.hkl

Supporting information file. DOI: 10.1107/S2056989020006398/wm5557Isup5.cml

CCDC references: 2003726, 2003725, 2003724

Additional supporting information:  crystallographic information; 3D view; checkCIF report

## Figures and Tables

**Table 1:** Hydrogen-bond geometry (Å, °) for (I)

*D*—H⋯*A*	*D*—H	H⋯*A*	*D*⋯*A*	*D*—H⋯*A*
N1—H11⋯O33	0.90 (2)	2.014 (19)	2.7968 (18)	144.8 (15)
N1—H11⋯O34	0.90 (2)	2.352 (19)	3.049 (2)	134.4 (14)
N1—H12⋯O31^i^	0.912 (19)	2.075 (19)	2.959 (2)	163.0 (17)
N1—H12⋯O32^i^	0.912 (19)	2.487 (18)	3.1576 (19)	130.7 (15)
O32—H32⋯O33	0.97 (3)	1.55 (3)	2.4676 (17)	157 (3)
C2—H2*B*⋯O35^ii^	0.97	2.51	3.313 (2)	140

Symmetry codes: (i) 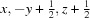; (ii) 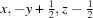.

**Table 2:** Hydrogen-bond geometry (Å, °) for (II)

*D*—H⋯*A*	*D*—H	H⋯*A*	*D*⋯*A*	*D*—H⋯*A*
N1—H11⋯O32	0.918 (16)	1.896 (16)	2.7769 (14)	160.2 (15)
N1—H12⋯O31^i^	0.920 (16)	1.902 (17)	2.7507 (14)	152.6 (15)
N1—H12⋯O34^i^	0.920 (16)	2.354 (16)	2.9588 (14)	123.1 (13)
O34—H34⋯O32^ii^	0.908 (17)	1.712 (17)	2.6102 (12)	170.0 (17)
C2—H2*A*⋯O33^iii^	0.97	2.54	3.4454 (15)	155
C5—H5*A*⋯O32^iv^	0.97	2.45	3.3849 (15)	163
C6—H6*B*⋯O31^v^	0.97	2.50	3.4259 (15)	159
C2—H2*B*⋯*Cg*1^vi^	0.97	2.65	3.6124 (14)	170

Symmetry codes: (i) 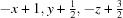; (ii) 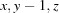; (iii) 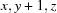; (iv) 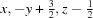; (v) 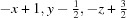; (vi) 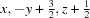.

**Table 3:** Hydrogen-bond geometry (Å, °) for (III) *Cg*1 represents the centroid of the ring (C21–C26).

*D*—H⋯*A*	*D*—H	H⋯*A*	*D*⋯*A*	*D*—H⋯*A*
N1—H11⋯O36	0.87 (4)	2.31 (4)	2.929 (4)	128 (3)
N1—H11⋯O35^i^	0.87 (4)	2.17 (4)	2.855 (4)	136 (3)
N1—H12⋯O41	0.92 (4)	1.83 (4)	2.740 (5)	169 (3)
O33—H33⋯O34^ii^	0.80 (4)	2.10 (4)	2.805 (3)	146 (3)
O34—H34⋯O31^ii^	0.81 (4)	2.07 (4)	2.806 (3)	151 (4)
O36—H36⋯O32^iii^	0.95 (4)	1.53 (4)	2.470 (3)	175 (3)
O41—H41⋯O31	0.96 (5)	1.82 (5)	2.771 (4)	178 (5)
O41—H42⋯O33^iv^	0.78 (5)	2.00 (5)	2.754 (4)	163 (5)
C25—H25⋯*Cg*1^v^	0.93	2.86	3.649 (5)	144

Symmetry codes: (i) 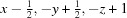; (ii) 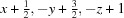; (iii) 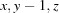; (iv) 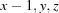; (v) 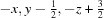.

**Table 4:** Experimental details

	(I)	(II)	(III)
Crystal data			
Chemical formula	C_10_H_14_FN_2_^+^·C_7_H_3_N_2_O_7_^−^	C_10_H_14_FN_2_^+^·C_2_HO_4_^−^	C_10_H_14_FN_2_^+^·C_4_H_5_O_6_^−^·H_2_O
*M*_r_	408.35	270.26	348.33
Crystal system, space group	Monoclinic, *P*2_1_/*c*	Monoclinic, *P*2_1_/*c*	Orthorhombic, *P*2_1_2_1_2_1_
Temperature (K)	293	293	293
*a*, *b*, *c* (Å)	10.6829 (6), 13.1701 (6), 13.5563 (7)	17.0606 (6), 5.7820 (2), 12.5815 (5)	7.0961 (4), 7.4967 (4), 30.757 (2)
α, β, γ (°)	90, 108.970 (5), 90	90, 102.761 (4), 90	90, 90, 90
*V* (Å^3^)	1803.71 (17)	1210.44 (8)	1636.19 (17)
*Z*	4	4	4
Radiation type	Mo *K*α	Mo *K*α	Mo *K*α
μ (mm^−1^)	0.13	0.12	0.12
Crystal size (mm)	0.50 × 0.44 × 0.34	0.34 × 0.34 × 0.28	0.40 × 0.22 × 0.10

Data collection			
Diffractometer	Oxford Diffraction Xcalibur with Sapphire CCD	Oxford Diffraction Xcalibur with Sapphire CCD	Oxford Diffraction Xcalibur with Sapphire CCD
Absorption correction	Multi-scan (*CrysAlis RED*; Oxford Diffraction, 2009 ▸)	Multi-scan (*CrysAlis RED*; Oxford Diffraction, 2009 ▸)	Multi-scan (*CrysAlis RED*; Oxford Diffraction, 2009 ▸)
*T*_min_, *T*_max_	0.874, 0.958	0.877, 0.966	0.904, 0.988
No. of measured, independent and observed [*I* > 2σ(*I*)] reflections	7194, 3905, 2845	4450, 2596, 2237	4553, 3036, 2347
*R*_int_	0.011	0.009	0.019
(sin θ/λ)_max_ (Å^−1^)	0.656	0.656	0.656

Refinement			
*R*[*F* ^2^ > 2σ(*F* ^2^)], *wR*(*F* ^2^), *S*	0.041, 0.108, 1.03	0.033, 0.089, 1.03	0.045, 0.085, 1.14
No. of reflections	3905	2596	3036
No. of parameters	271	182	238
H-atom treatment	H atoms treated by a mixture of independent and constrained refinement	H atoms treated by a mixture of independent and constrained refinement	H atoms treated by a mixture of independent and constrained refinement
Δρ_max_, Δρ_min_ (e Å^−3^)	0.29, −0.20	0.32, −0.14	0.18, −0.21
Absolute structure	–	–	Flack *x* determined using 683 quotients [(*I* ^+^)-(*I* ^-^)]/[(*I* ^+^)+(*I* ^-^)] (Parsons *et al.*, 2013 ▸)

Computer programs: *CrysAlis CCD* and *CrysAlis RED* (Oxford Diffraction, 2009 ▸), *SHELXT* (Sheldrick, 2015*a* ▸), *SHELXL2014* (Sheldrick, 2015*b* ▸) and *PLATON* (Spek, 2020 ▸).

## supplementary crystallographic information

### 4-(4-Fluorophenyl)piperazin-1-ium 2-hydroxy-3,5-dinitrobenzoate (I) Crystal data

**Table:**

C_10_H_14_FN_2_^+^·C_7_H_3_N_2_O_7_^−^	*F*(000) = 848
*M**_r_* = 408.35	*D*_x_ = 1.504 Mg m^−^^3^
Monoclinic, *P*2_1_/*c*	Mo *K*α radiation, λ = 0.71073 Å
*a* = 10.6829 (6) Å	Cell parameters from 3905 reflections
*b* = 13.1701 (6) Å	θ = 2.6–27.8°
*c* = 13.5563 (7) Å	µ = 0.13 mm^−^^1^
β = 108.970 (5)°	*T* = 293 K
*V* = 1803.71 (17) Å^3^	Block, yellow
*Z* = 4	0.50 × 0.44 × 0.34 mm

### 4-(4-Fluorophenyl)piperazin-1-ium 2-hydroxy-3,5-dinitrobenzoate (I) Data collection

**Table:**

Oxford Diffraction Xcalibur with Sapphire CCD diffractometer	3905 independent reflections
Radiation source: Enhance (Mo) X-ray Source	2845 reflections with *I* > 2σ(*I*)
Graphite monochromator	*R*_int_ = 0.011
ω scans	θ_max_ = 27.8°, θ_min_ = 2.6°
Absorption correction: multi-scan (CrysAlis RED; Oxford Diffraction, 2009)	*h* = −13→8
*T*_min_ = 0.874, *T*_max_ = 0.958	*k* = −17→12
7194 measured reflections	*l* = −11→17

### 4-(4-Fluorophenyl)piperazin-1-ium 2-hydroxy-3,5-dinitrobenzoate (I) Refinement

**Table:**

Refinement on *F*^2^	Primary atom site location: difference Fourier map
Least-squares matrix: full	Hydrogen site location: mixed
*R*[*F*^2^ > 2σ(*F*^2^)] = 0.041	H atoms treated by a mixture of independent and constrained refinement
*wR*(*F*^2^) = 0.108	*w* = 1/[σ^2^(*F*_o_^2^) + (0.0493*P*)^2^ + 0.4487*P*] where *P* = (*F*_o_^2^ + 2*F*_c_^2^)/3
*S* = 1.02	(Δ/σ)_max_ < 0.001
3905 reflections	Δρ_max_ = 0.29 e Å^−^^3^
271 parameters	Δρ_min_ = −0.20 e Å^−^^3^
0 restraints	

### 4-(4-Fluorophenyl)piperazin-1-ium 2-hydroxy-3,5-dinitrobenzoate (I) Special details

**Table:**

Geometry. All esds (except the esd in the dihedral angle between two l.s. planes) are estimated using the full covariance matrix. The cell esds are taken into account individually in the estimation of esds in distances, angles and torsion angles; correlations between esds in cell parameters are only used when they are defined by crystal symmetry. An approximate (isotropic) treatment of cell esds is used for estimating esds involving l.s. planes.

### 4-(4-Fluorophenyl)piperazin-1-ium 2-hydroxy-3,5-dinitrobenzoate (I) Fractional atomic coordinates and isotropic or equivalent isotropic displacement parameters (Å^2^)

**Table:**

	*x*	*y*	*z*	*U*_iso_*/*U*_eq_	
N1	0.23326 (15)	0.37624 (11)	0.56770 (10)	0.0410 (3)	
H11	0.2339 (18)	0.3081 (15)	0.5648 (13)	0.049*	
H12	0.1870 (18)	0.3961 (14)	0.6103 (14)	0.049*	
C2	0.16795 (18)	0.41660 (13)	0.46103 (12)	0.0458 (4)	
H2A	0.0754	0.3969	0.4369	0.055*	
H2B	0.2098	0.3883	0.4135	0.055*	
C3	0.17888 (16)	0.53003 (12)	0.46196 (13)	0.0446 (4)	
H3A	0.1373	0.5561	0.3920	0.054*	
H3B	0.1328	0.5583	0.5066	0.054*	
N4	0.31711 (13)	0.56110 (10)	0.49976 (10)	0.0386 (3)	
C5	0.38008 (18)	0.52566 (14)	0.60598 (13)	0.0507 (4)	
H5A	0.3366	0.5558	0.6515	0.061*	
H5B	0.4721	0.5466	0.6302	0.061*	
C6	0.37196 (18)	0.41196 (14)	0.61064 (15)	0.0527 (4)	
H6A	0.4238	0.3820	0.5712	0.063*	
H6B	0.4093	0.3898	0.6825	0.063*	
C21	0.34479 (15)	0.66117 (12)	0.47504 (12)	0.0369 (3)	
C22	0.29655 (18)	0.69322 (13)	0.37175 (13)	0.0481 (4)	
H22	0.2418	0.6502	0.3218	0.058*	
C23	0.3279 (2)	0.78703 (14)	0.34172 (15)	0.0545 (5)	
H23	0.2948	0.8074	0.2724	0.065*	
C24	0.40826 (18)	0.84938 (13)	0.41541 (16)	0.0505 (4)	
F24	0.44076 (13)	0.94182 (8)	0.38489 (10)	0.0748 (4)	
C25	0.45600 (18)	0.82219 (13)	0.51707 (16)	0.0533 (5)	
H25	0.5101	0.8664	0.5660	0.064*	
C26	0.42392 (17)	0.72812 (13)	0.54787 (13)	0.0464 (4)	
H26	0.4557	0.7097	0.6179	0.056*	
C37	0.15341 (16)	0.07140 (13)	0.28960 (12)	0.0422 (4)	
O31	0.11601 (13)	0.02065 (10)	0.21079 (8)	0.0549 (3)	
O32	0.19930 (15)	0.16315 (11)	0.28736 (10)	0.0644 (4)	
H32	0.224 (3)	0.1886 (19)	0.358 (2)	0.097*	
C31	0.15560 (14)	0.03293 (11)	0.39367 (11)	0.0336 (3)	
C32	0.21010 (14)	0.09577 (11)	0.48464 (11)	0.0328 (3)	
O33	0.24279 (12)	0.18739 (8)	0.47606 (8)	0.0461 (3)	
C33	0.22155 (15)	0.04718 (11)	0.58166 (11)	0.0346 (3)	
C34	0.17610 (15)	−0.04917 (12)	0.58678 (12)	0.0384 (4)	
H34	0.1844	−0.0782	0.6511	0.046*	
C35	0.11827 (15)	−0.10288 (12)	0.49668 (12)	0.0379 (3)	
C36	0.10914 (14)	−0.06281 (12)	0.39986 (12)	0.0365 (3)	
H36	0.0717	−0.1008	0.3396	0.044*	
N33	0.28212 (14)	0.09929 (11)	0.68062 (10)	0.0456 (3)	
O34	0.33859 (15)	0.17925 (10)	0.68299 (10)	0.0654 (4)	
O35	0.2769 (2)	0.05901 (14)	0.75965 (10)	0.0944 (6)	
N35	0.06646 (15)	−0.20338 (11)	0.50384 (13)	0.0497 (4)	
O36	0.09063 (16)	−0.24215 (11)	0.58996 (12)	0.0768 (5)	
O37	−0.00086 (15)	−0.24444 (10)	0.42356 (12)	0.0668 (4)	

### 4-(4-Fluorophenyl)piperazin-1-ium 2-hydroxy-3,5-dinitrobenzoate (I) Atomic displacement parameters (Å^2^)

**Table:**

	*U*^11^	*U*^22^	*U*^33^	*U*^12^	*U*^13^	*U*^23^
N1	0.0566 (9)	0.0317 (7)	0.0400 (7)	−0.0031 (6)	0.0228 (6)	−0.0008 (6)
C2	0.0570 (10)	0.0409 (9)	0.0380 (8)	−0.0130 (8)	0.0132 (7)	−0.0001 (7)
C3	0.0416 (9)	0.0372 (9)	0.0506 (9)	−0.0068 (7)	0.0088 (7)	0.0042 (7)
N4	0.0405 (7)	0.0348 (7)	0.0378 (7)	−0.0064 (6)	0.0091 (5)	0.0011 (5)
C5	0.0513 (10)	0.0503 (10)	0.0426 (9)	−0.0115 (8)	0.0044 (8)	0.0038 (8)
C6	0.0504 (10)	0.0503 (11)	0.0544 (10)	0.0019 (8)	0.0131 (8)	0.0131 (9)
C21	0.0373 (8)	0.0338 (8)	0.0422 (8)	−0.0041 (6)	0.0166 (6)	−0.0040 (6)
C22	0.0613 (11)	0.0398 (9)	0.0434 (9)	−0.0120 (8)	0.0171 (8)	−0.0032 (7)
C23	0.0682 (12)	0.0464 (10)	0.0528 (10)	−0.0056 (9)	0.0251 (9)	0.0068 (8)
C24	0.0522 (10)	0.0331 (9)	0.0740 (13)	−0.0064 (8)	0.0313 (9)	0.0020 (8)
F24	0.0864 (9)	0.0408 (6)	0.1059 (10)	−0.0168 (6)	0.0432 (7)	0.0086 (6)
C25	0.0485 (10)	0.0404 (10)	0.0705 (12)	−0.0149 (8)	0.0187 (9)	−0.0135 (9)
C26	0.0464 (9)	0.0439 (10)	0.0466 (9)	−0.0088 (8)	0.0121 (7)	−0.0059 (7)
C37	0.0451 (9)	0.0506 (10)	0.0317 (8)	0.0017 (8)	0.0138 (7)	0.0028 (7)
O31	0.0654 (8)	0.0674 (8)	0.0314 (6)	−0.0048 (7)	0.0151 (5)	−0.0039 (6)
O32	0.1018 (11)	0.0559 (8)	0.0392 (7)	−0.0167 (8)	0.0279 (7)	0.0062 (6)
C31	0.0340 (7)	0.0384 (8)	0.0304 (7)	0.0037 (6)	0.0132 (6)	0.0013 (6)
C32	0.0346 (7)	0.0330 (8)	0.0337 (7)	0.0025 (6)	0.0152 (6)	0.0016 (6)
O33	0.0662 (8)	0.0337 (6)	0.0416 (6)	−0.0063 (5)	0.0219 (5)	0.0001 (5)
C33	0.0364 (8)	0.0376 (8)	0.0312 (7)	0.0025 (6)	0.0126 (6)	−0.0002 (6)
C34	0.0393 (8)	0.0419 (9)	0.0370 (8)	0.0051 (7)	0.0165 (7)	0.0095 (7)
C35	0.0362 (8)	0.0323 (8)	0.0476 (9)	0.0007 (6)	0.0169 (7)	0.0039 (7)
C36	0.0341 (8)	0.0374 (8)	0.0387 (8)	0.0005 (6)	0.0130 (6)	−0.0037 (6)
N33	0.0534 (8)	0.0503 (9)	0.0327 (7)	0.0013 (7)	0.0133 (6)	0.0002 (6)
O34	0.0900 (11)	0.0529 (8)	0.0464 (7)	−0.0153 (7)	0.0128 (7)	−0.0104 (6)
O35	0.1494 (17)	0.0992 (13)	0.0325 (7)	−0.0423 (11)	0.0265 (8)	0.0008 (7)
N35	0.0475 (8)	0.0385 (8)	0.0662 (10)	−0.0035 (6)	0.0228 (7)	0.0050 (7)
O36	0.0962 (11)	0.0551 (9)	0.0759 (10)	−0.0183 (8)	0.0235 (8)	0.0238 (8)
O37	0.0731 (9)	0.0487 (8)	0.0777 (10)	−0.0215 (7)	0.0232 (8)	−0.0112 (7)

### 4-(4-Fluorophenyl)piperazin-1-ium 2-hydroxy-3,5-dinitrobenzoate (I) Geometric parameters (Å, º)

**Table:**

N1—C6	1.481 (2)	C24—F24	1.3664 (19)
N1—C2	1.485 (2)	C25—C26	1.386 (2)
N1—H11	0.898 (19)	C25—H25	0.9300
N1—H12	0.912 (19)	C26—H26	0.9300
C2—C3	1.498 (2)	C37—O31	1.2125 (19)
C2—H2A	0.9700	C37—O32	1.308 (2)
C2—H2B	0.9700	C37—C31	1.492 (2)
C3—N4	1.456 (2)	O32—H32	0.97 (3)
C3—H3A	0.9700	C31—C36	1.368 (2)
C3—H3B	0.9700	C31—C32	1.441 (2)
N4—C21	1.4147 (19)	C32—O33	1.2719 (18)
N4—C5	1.454 (2)	C32—C33	1.4318 (19)
C5—C6	1.502 (2)	C33—C34	1.368 (2)
C5—H5A	0.9700	C33—N33	1.4578 (19)
C5—H5B	0.9700	C34—C35	1.372 (2)
C6—H6A	0.9700	C34—H34	0.9300
C6—H6B	0.9700	C35—C36	1.388 (2)
C21—C26	1.387 (2)	C35—N35	1.450 (2)
C21—C22	1.391 (2)	C36—H36	0.9300
C22—C23	1.376 (2)	N33—O34	1.2089 (19)
C22—H22	0.9300	N33—O35	1.2129 (18)
C23—C24	1.361 (3)	N35—O37	1.2187 (19)
C23—H23	0.9300	N35—O36	1.2224 (19)
C24—C25	1.353 (3)		
			
C6—N1—C2	111.24 (13)	C24—C23—H23	120.6
C6—N1—H11	108.3 (12)	C22—C23—H23	120.6
C2—N1—H11	108.8 (11)	C25—C24—C23	121.80 (16)
C6—N1—H12	109.8 (11)	C25—C24—F24	119.70 (17)
C2—N1—H12	109.5 (11)	C23—C24—F24	118.50 (17)
H11—N1—H12	109.1 (16)	C24—C25—C26	119.69 (16)
N1—C2—C3	109.76 (13)	C24—C25—H25	120.2
N1—C2—H2A	109.7	C26—C25—H25	120.2
C3—C2—H2A	109.7	C25—C26—C21	120.47 (16)
N1—C2—H2B	109.7	C25—C26—H26	119.8
C3—C2—H2B	109.7	C21—C26—H26	119.8
H2A—C2—H2B	108.2	O31—C37—O32	120.48 (15)
N4—C3—C2	110.53 (14)	O31—C37—C31	123.05 (16)
N4—C3—H3A	109.5	O32—C37—C31	116.42 (14)
C2—C3—H3A	109.5	C37—O32—H32	106.3 (15)
N4—C3—H3B	109.5	C36—C31—C32	122.05 (13)
C2—C3—H3B	109.5	C36—C31—C37	118.46 (13)
H3A—C3—H3B	108.1	C32—C31—C37	119.46 (13)
C21—N4—C5	117.95 (13)	O33—C32—C33	124.33 (13)
C21—N4—C3	116.41 (13)	O33—C32—C31	120.87 (13)
C5—N4—C3	110.38 (13)	C33—C32—C31	114.80 (13)
N4—C5—C6	110.31 (14)	C34—C33—C32	122.36 (13)
N4—C5—H5A	109.6	C34—C33—N33	116.65 (13)
C6—C5—H5A	109.6	C32—C33—N33	120.98 (13)
N4—C5—H5B	109.6	C33—C34—C35	119.82 (14)
C6—C5—H5B	109.6	C33—C34—H34	120.1
H5A—C5—H5B	108.1	C35—C34—H34	120.1
N1—C6—C5	111.34 (15)	C34—C35—C36	121.23 (14)
N1—C6—H6A	109.4	C34—C35—N35	118.82 (14)
C5—C6—H6A	109.4	C36—C35—N35	119.95 (14)
N1—C6—H6B	109.4	C31—C36—C35	119.54 (14)
C5—C6—H6B	109.4	C31—C36—H36	120.2
H6A—C6—H6B	108.0	C35—C36—H36	120.2
C26—C21—C22	117.66 (15)	O34—N33—O35	121.56 (15)
C26—C21—N4	123.34 (14)	O34—N33—C33	120.20 (13)
C22—C21—N4	118.92 (14)	O35—N33—C33	118.22 (15)
C23—C22—C21	121.61 (16)	O37—N35—O36	123.20 (15)
C23—C22—H22	119.2	O37—N35—C35	118.20 (15)
C21—C22—H22	119.2	O36—N35—C35	118.59 (15)
C24—C23—C22	118.74 (17)		
			
C6—N1—C2—C3	−54.64 (19)	O32—C37—C31—C32	−1.7 (2)
N1—C2—C3—N4	58.34 (18)	C36—C31—C32—O33	−174.27 (14)
C2—C3—N4—C21	160.93 (13)	C37—C31—C32—O33	7.8 (2)
C2—C3—N4—C5	−61.10 (18)	C36—C31—C32—C33	4.9 (2)
C21—N4—C5—C6	−163.70 (14)	C37—C31—C32—C33	−173.01 (13)
C3—N4—C5—C6	59.05 (19)	O33—C32—C33—C34	175.03 (14)
C2—N1—C6—C5	53.7 (2)	C31—C32—C33—C34	−4.1 (2)
N4—C5—C6—N1	−55.5 (2)	O33—C32—C33—N33	−4.5 (2)
C5—N4—C21—C26	−2.9 (2)	C31—C32—C33—N33	176.36 (13)
C3—N4—C21—C26	131.79 (17)	C32—C33—C34—C35	0.6 (2)
C5—N4—C21—C22	173.56 (16)	N33—C33—C34—C35	−179.81 (14)
C3—N4—C21—C22	−51.7 (2)	C33—C34—C35—C36	2.4 (2)
C26—C21—C22—C23	1.3 (3)	C33—C34—C35—N35	−177.75 (14)
N4—C21—C22—C23	−175.35 (16)	C32—C31—C36—C35	−2.2 (2)
C21—C22—C23—C24	0.1 (3)	C37—C31—C36—C35	175.71 (14)
C22—C23—C24—C25	−1.1 (3)	C34—C35—C36—C31	−1.6 (2)
C22—C23—C24—F24	178.99 (16)	N35—C35—C36—C31	178.55 (14)
C23—C24—C25—C26	0.6 (3)	C34—C33—N33—O34	171.04 (15)
F24—C24—C25—C26	−179.47 (16)	C32—C33—N33—O34	−9.4 (2)
C24—C25—C26—C21	0.9 (3)	C34—C33—N33—O35	−7.2 (2)
C22—C21—C26—C25	−1.8 (2)	C32—C33—N33—O35	172.40 (16)
N4—C21—C26—C25	174.71 (15)	C34—C35—N35—O37	169.81 (15)
O31—C37—C31—C36	−2.3 (2)	C36—C35—N35—O37	−10.4 (2)
O32—C37—C31—C36	−179.71 (15)	C34—C35—N35—O36	−9.1 (2)
O31—C37—C31—C32	175.74 (15)	C36—C35—N35—O36	170.72 (15)

### 4-(4-Fluorophenyl)piperazin-1-ium 2-hydroxy-3,5-dinitrobenzoate (I) Hydrogen-bond geometry (Å, º)

**Table:**

*D*—H···*A*	*D*—H	H···*A*	*D*···*A*	*D*—H···*A*
N1—H11···O33	0.90 (2)	2.014 (19)	2.7968 (18)	144.8 (15)
N1—H11···O34	0.90 (2)	2.352 (19)	3.049 (2)	134.4 (14)
N1—H12···O31^i^	0.912 (19)	2.075 (19)	2.959 (2)	163.0 (17)
N1—H12···O32^i^	0.912 (19)	2.487 (18)	3.1576 (19)	130.7 (15)
O32—H32···O33	0.97 (3)	1.55 (3)	2.4676 (17)	157 (3)
C2—H2*B*···O35^ii^	0.97	2.51	3.313 (2)	140

Symmetry codes: (i) *x*, −*y*+1/2, *z*+1/2; (ii) *x*, −*y*+1/2, *z*−1/2.

### 4-(4-Fluorophenyl)piperazin-1-ium hydrogen oxalate (II) Crystal data

**Table:**

C_10_H_14_FN_2_^+^·C_2_HO_4_^−^	*F*(000) = 568
*M**_r_* = 270.26	*D*_x_ = 1.483 Mg m^−^^3^
Monoclinic, *P*2_1_/*c*	Mo *K*α radiation, λ = 0.71073 Å
*a* = 17.0606 (6) Å	Cell parameters from 2596 reflections
*b* = 5.7820 (2) Å	θ = 3.3–27.8°
*c* = 12.5815 (5) Å	µ = 0.12 mm^−^^1^
β = 102.761 (4)°	*T* = 293 K
*V* = 1210.44 (8) Å^3^	Block, colourless
*Z* = 4	0.34 × 0.34 × 0.28 mm

### 4-(4-Fluorophenyl)piperazin-1-ium hydrogen oxalate (II) Data collection

**Table:**

Oxford Diffraction Xcalibur with Sapphire CCD diffractometer	2596 independent reflections
Radiation source: Enhance (Mo) X-ray Source	2237 reflections with *I* > 2σ(*I*)
Graphite monochromator	*R*_int_ = 0.009
ω scans	θ_max_ = 27.8°, θ_min_ = 3.3°
Absorption correction: multi-scan (CrysAlis RED; Oxford Diffraction, 2009)	*h* = −16→22
*T*_min_ = 0.877, *T*_max_ = 0.966	*k* = −7→5
4450 measured reflections	*l* = −16→9

### 4-(4-Fluorophenyl)piperazin-1-ium hydrogen oxalate (II) Refinement

**Table:**

Refinement on *F*^2^	Hydrogen site location: mixed
Least-squares matrix: full	H atoms treated by a mixture of independent and constrained refinement
*R*[*F*^2^ > 2σ(*F*^2^)] = 0.033	*w* = 1/[σ^2^(*F*_o_^2^) + (0.0443*P*)^2^ + 0.346*P*] where *P* = (*F*_o_^2^ + 2*F*_c_^2^)/3
*wR*(*F*^2^) = 0.089	(Δ/σ)_max_ < 0.001
*S* = 1.03	Δρ_max_ = 0.32 e Å^−^^3^
2596 reflections	Δρ_min_ = −0.14 e Å^−^^3^
182 parameters	Extinction correction: SHELXL, Fc^*^=kFc[1+0.001xFc^2^λ^3^/sin(2θ)]^-1/4^
0 restraints	Extinction coefficient: 0.0084 (12)
Primary atom site location: difference Fourier map	

### 4-(4-Fluorophenyl)piperazin-1-ium hydrogen oxalate (II) Special details

**Table:**

Geometry. All esds (except the esd in the dihedral angle between two l.s. planes) are estimated using the full covariance matrix. The cell esds are taken into account individually in the estimation of esds in distances, angles and torsion angles; correlations between esds in cell parameters are only used when they are defined by crystal symmetry. An approximate (isotropic) treatment of cell esds is used for estimating esds involving l.s. planes.

### 4-(4-Fluorophenyl)piperazin-1-ium hydrogen oxalate (II) Fractional atomic coordinates and isotropic or equivalent isotropic displacement parameters (Å^2^)

**Table:**

	*x*	*y*	*z*	*U*_iso_*/*U*_eq_	
N1	0.34076 (6)	0.76074 (19)	0.62909 (9)	0.0310 (2)	
H11	0.3629 (9)	0.731 (3)	0.7011 (13)	0.037*	
H12	0.3813 (9)	0.831 (3)	0.6035 (12)	0.037*	
C2	0.27227 (7)	0.9240 (2)	0.62039 (10)	0.0316 (3)	
H2A	0.2911	1.0681	0.6566	0.038*	
H2B	0.2324	0.8584	0.6559	0.038*	
C3	0.23469 (8)	0.9699 (2)	0.50098 (10)	0.0324 (3)	
H3A	0.1896	1.0749	0.4950	0.039*	
H3B	0.2739	1.0411	0.4660	0.039*	
N4	0.20732 (6)	0.75101 (17)	0.44718 (8)	0.0268 (2)	
C5	0.27693 (7)	0.5982 (2)	0.45117 (10)	0.0319 (3)	
H5A	0.3154	0.6735	0.4163	0.038*	
H5B	0.2595	0.4561	0.4120	0.038*	
C6	0.31618 (8)	0.5431 (2)	0.56812 (11)	0.0347 (3)	
H6A	0.2788	0.4590	0.6017	0.042*	
H6B	0.3629	0.4461	0.5705	0.042*	
C21	0.15689 (7)	0.7661 (2)	0.34045 (9)	0.0276 (3)	
C22	0.10736 (8)	0.5776 (2)	0.30382 (11)	0.0364 (3)	
H22	0.1070	0.4520	0.3499	0.044*	
C23	0.05858 (8)	0.5743 (3)	0.19977 (12)	0.0424 (3)	
H23	0.0262	0.4472	0.1753	0.051*	
C24	0.05932 (8)	0.7625 (3)	0.13391 (11)	0.0418 (3)	
F24	0.01133 (7)	0.75923 (18)	0.03205 (7)	0.0688 (3)	
C25	0.10587 (9)	0.9541 (3)	0.16709 (11)	0.0418 (3)	
H25	0.1044	1.0806	0.1210	0.050*	
C26	0.15542 (8)	0.9549 (2)	0.27143 (10)	0.0344 (3)	
H26	0.1877	1.0826	0.2950	0.041*	
C31	0.47968 (7)	0.58033 (19)	0.86963 (9)	0.0239 (2)	
C32	0.43856 (7)	0.3385 (2)	0.84980 (9)	0.0258 (2)	
O31	0.55249 (5)	0.58853 (15)	0.91087 (7)	0.0319 (2)	
O32	0.43316 (5)	0.74960 (14)	0.84049 (7)	0.0315 (2)	
O33	0.36759 (5)	0.31730 (17)	0.81152 (8)	0.0417 (2)	
O34	0.48994 (5)	0.16802 (15)	0.87815 (7)	0.0320 (2)	
H34	0.4648 (10)	0.029 (3)	0.8663 (13)	0.048*	

### 4-(4-Fluorophenyl)piperazin-1-ium hydrogen oxalate (II) Atomic displacement parameters (Å^2^)

**Table:**

	*U*^11^	*U*^22^	*U*^33^	*U*^12^	*U*^13^	*U*^23^
N1	0.0245 (5)	0.0331 (6)	0.0310 (5)	−0.0043 (4)	−0.0037 (4)	0.0048 (4)
C2	0.0321 (6)	0.0295 (6)	0.0305 (6)	−0.0019 (5)	0.0008 (5)	−0.0036 (5)
C3	0.0355 (6)	0.0246 (6)	0.0324 (6)	0.0032 (5)	−0.0025 (5)	−0.0014 (5)
N4	0.0250 (5)	0.0250 (5)	0.0273 (5)	0.0024 (4)	−0.0007 (4)	−0.0001 (4)
C5	0.0302 (6)	0.0289 (6)	0.0345 (6)	0.0046 (5)	0.0025 (5)	−0.0019 (5)
C6	0.0301 (6)	0.0278 (6)	0.0411 (7)	0.0033 (5)	−0.0032 (5)	0.0027 (5)
C21	0.0245 (5)	0.0300 (6)	0.0265 (6)	0.0042 (5)	0.0019 (4)	−0.0010 (5)
C22	0.0349 (7)	0.0339 (7)	0.0363 (7)	−0.0028 (5)	−0.0008 (5)	0.0016 (5)
C23	0.0370 (7)	0.0411 (8)	0.0424 (7)	−0.0024 (6)	−0.0058 (6)	−0.0082 (6)
C24	0.0402 (7)	0.0471 (8)	0.0303 (6)	0.0124 (6)	−0.0093 (5)	−0.0052 (6)
F24	0.0811 (7)	0.0650 (6)	0.0406 (5)	0.0116 (5)	−0.0291 (5)	−0.0051 (5)
C25	0.0503 (8)	0.0382 (7)	0.0326 (7)	0.0108 (6)	−0.0001 (6)	0.0061 (6)
C26	0.0363 (6)	0.0300 (6)	0.0338 (6)	0.0018 (5)	0.0011 (5)	0.0003 (5)
C31	0.0273 (5)	0.0224 (5)	0.0215 (5)	0.0006 (4)	0.0042 (4)	−0.0009 (4)
C32	0.0269 (6)	0.0248 (6)	0.0241 (5)	−0.0004 (4)	0.0026 (4)	−0.0004 (4)
O31	0.0246 (4)	0.0274 (4)	0.0405 (5)	−0.0014 (3)	0.0006 (3)	−0.0036 (4)
O32	0.0324 (5)	0.0224 (4)	0.0360 (5)	0.0037 (3)	−0.0002 (4)	0.0008 (3)
O33	0.0272 (5)	0.0349 (5)	0.0560 (6)	−0.0030 (4)	−0.0057 (4)	−0.0029 (4)
O34	0.0292 (4)	0.0200 (4)	0.0442 (5)	−0.0003 (3)	0.0026 (4)	0.0000 (4)

### 4-(4-Fluorophenyl)piperazin-1-ium hydrogen oxalate (II) Geometric parameters (Å, º)

**Table:**

N1—C6	1.4854 (16)	C21—C26	1.3917 (18)
N1—C2	1.4872 (16)	C21—C22	1.3935 (17)
N1—H11	0.918 (16)	C22—C23	1.3875 (18)
N1—H12	0.920 (16)	C22—H22	0.9300
C2—C3	1.5208 (16)	C23—C24	1.370 (2)
C2—H2A	0.9700	C23—H23	0.9300
C2—H2B	0.9700	C24—F24	1.3600 (15)
C3—N4	1.4622 (15)	C24—C25	1.373 (2)
C3—H3A	0.9700	C25—C26	1.3956 (17)
C3—H3B	0.9700	C25—H25	0.9300
N4—C21	1.4284 (14)	C26—H26	0.9300
N4—C5	1.4723 (15)	C31—O31	1.2368 (13)
C5—C6	1.5098 (17)	C31—O32	1.2625 (13)
C5—H5A	0.9700	C31—C32	1.5597 (16)
C5—H5B	0.9700	C32—O33	1.2064 (14)
C6—H6A	0.9700	C32—O34	1.3148 (14)
C6—H6B	0.9700	O34—H34	0.908 (18)
			
C6—N1—C2	111.85 (9)	C5—C6—H6A	109.7
C6—N1—H11	110.9 (9)	N1—C6—H6B	109.7
C2—N1—H11	109.8 (9)	C5—C6—H6B	109.7
C6—N1—H12	110.0 (9)	H6A—C6—H6B	108.2
C2—N1—H12	109.5 (9)	C26—C21—C22	118.75 (11)
H11—N1—H12	104.5 (13)	C26—C21—N4	123.95 (11)
N1—C2—C3	109.65 (10)	C22—C21—N4	117.30 (11)
N1—C2—H2A	109.7	C23—C22—C21	121.10 (13)
C3—C2—H2A	109.7	C23—C22—H22	119.4
N1—C2—H2B	109.7	C21—C22—H22	119.4
C3—C2—H2B	109.7	C24—C23—C22	118.41 (13)
H2A—C2—H2B	108.2	C24—C23—H23	120.8
N4—C3—C2	109.16 (10)	C22—C23—H23	120.8
N4—C3—H3A	109.8	F24—C24—C23	118.36 (13)
C2—C3—H3A	109.8	F24—C24—C25	119.02 (13)
N4—C3—H3B	109.8	C23—C24—C25	122.63 (12)
C2—C3—H3B	109.8	C24—C25—C26	118.56 (13)
H3A—C3—H3B	108.3	C24—C25—H25	120.7
C21—N4—C3	116.55 (9)	C26—C25—H25	120.7
C21—N4—C5	112.49 (9)	C21—C26—C25	120.53 (12)
C3—N4—C5	109.32 (9)	C21—C26—H26	119.7
N4—C5—C6	109.92 (10)	C25—C26—H26	119.7
N4—C5—H5A	109.7	O31—C31—O32	126.93 (11)
C6—C5—H5A	109.7	O31—C31—C32	118.43 (10)
N4—C5—H5B	109.7	O32—C31—C32	114.64 (9)
C6—C5—H5B	109.7	O33—C32—O34	125.62 (11)
H5A—C5—H5B	108.2	O33—C32—C31	122.08 (10)
N1—C6—C5	109.77 (10)	O34—C32—C31	112.29 (9)
N1—C6—H6A	109.7	C32—O34—H34	110.9 (10)
			
C6—N1—C2—C3	−55.37 (14)	N4—C21—C22—C23	−177.69 (12)
N1—C2—C3—N4	58.86 (13)	C21—C22—C23—C24	−0.9 (2)
C2—C3—N4—C21	168.31 (10)	C22—C23—C24—F24	−179.84 (13)
C2—C3—N4—C5	−62.77 (13)	C22—C23—C24—C25	−0.5 (2)
C21—N4—C5—C6	−166.31 (10)	F24—C24—C25—C26	−179.45 (13)
C3—N4—C5—C6	62.56 (13)	C23—C24—C25—C26	1.2 (2)
C2—N1—C6—C5	54.78 (14)	C22—C21—C26—C25	−0.68 (19)
N4—C5—C6—N1	−57.70 (14)	N4—C21—C26—C25	178.36 (12)
C3—N4—C21—C26	23.67 (17)	C24—C25—C26—C21	−0.6 (2)
C5—N4—C21—C26	−103.72 (14)	O31—C31—C32—O33	178.81 (11)
C3—N4—C21—C22	−157.27 (11)	O32—C31—C32—O33	−1.35 (16)
C5—N4—C21—C22	75.34 (14)	O31—C31—C32—O34	−1.92 (14)
C26—C21—C22—C23	1.42 (19)	O32—C31—C32—O34	177.92 (10)

### 4-(4-Fluorophenyl)piperazin-1-ium hydrogen oxalate (II) Hydrogen-bond geometry (Å, º)

**Table:**

*D*—H···*A*	*D*—H	H···*A*	*D*···*A*	*D*—H···*A*
N1—H11···O32	0.918 (16)	1.896 (16)	2.7769 (14)	160.2 (15)
N1—H12···O31^i^	0.920 (16)	1.902 (17)	2.7507 (14)	152.6 (15)
N1—H12···O34^i^	0.920 (16)	2.354 (16)	2.9588 (14)	123.1 (13)
O34—H34···O32^ii^	0.908 (17)	1.712 (17)	2.6102 (12)	170.0 (17)
C2—H2*A*···O33^iii^	0.97	2.54	3.4454 (15)	155
C5—H5*A*···O32^iv^	0.97	2.45	3.3849 (15)	163
C6—H6*B*···O31^v^	0.97	2.50	3.4259 (15)	159
C2—H2*B*···*Cg*1^vi^	0.97	2.65	3.6124 (14)	170
C23—H23···*Cg*1^vii^	0.93	2.94	3.5865 (16)	128

Symmetry codes: (i) −*x*+1, *y*+1/2, −*z*+3/2; (ii) *x*, *y*−1, *z*; (iii) *x*, *y*+1, *z*; (iv) *x*, −*y*+3/2, *z*−1/2; (v) −*x*+1, *y*−1/2, −*z*+3/2; (vi) *x*, −*y*+3/2, *z*+1/2; (vii) −*x*, *y*−1/2, −*z*+1/2.

### 4-(4-Fluorophenyl)piperazin-1-ium hydrogen (2R,3R)-tartrate monohydrate (III) Crystal data

**Table:**

C_10_H_14_FN_2_^+^·C_4_H_5_O_6_^−^·H_2_O	*D*_x_ = 1.414 Mg m^−^^3^
*M**_r_* = 348.33	Mo *K*α radiation, λ = 0.71073 Å
Orthorhombic, *P*2_1_2_1_2_1_	Cell parameters from 3036 reflections
*a* = 7.0961 (4) Å	θ = 2.7–27.8°
*b* = 7.4967 (4) Å	µ = 0.12 mm^−^^1^
*c* = 30.757 (2) Å	*T* = 293 K
*V* = 1636.19 (17) Å^3^	Needle, yellow
*Z* = 4	0.40 × 0.22 × 0.10 mm
*F*(000) = 736	

### 4-(4-Fluorophenyl)piperazin-1-ium hydrogen (2R,3R)-tartrate monohydrate (III) Data collection

**Table:**

Oxford Diffraction Xcalibur with Sapphire CCD diffractometer	3036 independent reflections
Radiation source: Enhance (Mo) X-ray Source	2347 reflections with *I* > 2σ(*I*)
Graphite monochromator	*R*_int_ = 0.019
ω scans	θ_max_ = 27.8°, θ_min_ = 2.7°
Absorption correction: multi-scan (CrysAlis RED; Oxford Diffraction, 2009)	*h* = −8→7
*T*_min_ = 0.904, *T*_max_ = 0.988	*k* = −7→9
4553 measured reflections	*l* = −39→20

### 4-(4-Fluorophenyl)piperazin-1-ium hydrogen (2R,3R)-tartrate monohydrate (III) Refinement

**Table:**

Refinement on *F*^2^	Primary atom site location: difference Fourier map
Least-squares matrix: full	Hydrogen site location: mixed
*R*[*F*^2^ > 2σ(*F*^2^)] = 0.045	H atoms treated by a mixture of independent and constrained refinement
*wR*(*F*^2^) = 0.085	*w* = 1/[σ^2^(*F*_o_^2^) + (0.0206*P*)^2^ + 0.5183*P*] where *P* = (*F*_o_^2^ + 2*F*_c_^2^)/3
*S* = 1.14	(Δ/σ)_max_ < 0.001
3036 reflections	Δρ_max_ = 0.18 e Å^−^^3^
238 parameters	Δρ_min_ = −0.21 e Å^−^^3^
0 restraints	Absolute structure: Flack *x* determined using 683 quotients [(*I*^+^)-(*I*^-^)]/[(*I*^+^)+(*I*^-^)] (Parsons *et al.*, 2013)

### 4-(4-Fluorophenyl)piperazin-1-ium hydrogen (2R,3R)-tartrate monohydrate (III) Special details

**Table:**

Geometry. All esds (except the esd in the dihedral angle between two l.s. planes) are estimated using the full covariance matrix. The cell esds are taken into account individually in the estimation of esds in distances, angles and torsion angles; correlations between esds in cell parameters are only used when they are defined by crystal symmetry. An approximate (isotropic) treatment of cell esds is used for estimating esds involving l.s. planes.

### 4-(4-Fluorophenyl)piperazin-1-ium hydrogen (2R,3R)-tartrate monohydrate (III) Fractional atomic coordinates and isotropic or equivalent isotropic displacement parameters (Å^2^)

**Table:**

	*x*	*y*	*z*	*U*_iso_*/*U*_eq_	
N1	0.6296 (4)	0.2270 (5)	0.60097 (11)	0.0454 (8)	
H11	0.688 (5)	0.200 (5)	0.5771 (12)	0.054*	
H12	0.623 (5)	0.349 (5)	0.5995 (12)	0.054*	
C2	0.7310 (4)	0.1802 (5)	0.64079 (12)	0.0464 (9)	
H2A	0.8493	0.2450	0.6418	0.056*	
H2B	0.7597	0.0537	0.6405	0.056*	
C3	0.6174 (5)	0.2235 (5)	0.68063 (11)	0.0424 (9)	
H3A	0.6852	0.1847	0.7063	0.051*	
H3B	0.5998	0.3516	0.6826	0.051*	
N4	0.4346 (3)	0.1361 (4)	0.67912 (8)	0.0359 (7)	
C5	0.3323 (5)	0.1901 (5)	0.64017 (11)	0.0464 (9)	
H5A	0.3106	0.3178	0.6410	0.056*	
H5B	0.2107	0.1311	0.6396	0.056*	
C6	0.4402 (5)	0.1435 (6)	0.59966 (11)	0.0521 (10)	
H6A	0.4530	0.0150	0.5974	0.063*	
H6B	0.3720	0.1855	0.5743	0.063*	
C21	0.3285 (5)	0.1373 (4)	0.71803 (10)	0.0350 (7)	
C22	0.3867 (5)	0.2237 (5)	0.75550 (11)	0.0439 (9)	
H22	0.4981	0.2890	0.7551	0.053*	
C23	0.2824 (6)	0.2149 (5)	0.79359 (12)	0.0554 (11)	
H23	0.3243	0.2707	0.8188	0.066*	
C24	0.1175 (6)	0.1230 (5)	0.79317 (12)	0.0541 (10)	
F24	0.0137 (4)	0.1146 (4)	0.83031 (7)	0.0923 (9)	
C25	0.0551 (5)	0.0358 (6)	0.75739 (12)	0.0547 (11)	
H25	−0.0575	−0.0276	0.7582	0.066*	
C26	0.1605 (5)	0.0422 (5)	0.71956 (12)	0.0456 (9)	
H26	0.1185	−0.0178	0.6949	0.055*	
C31	0.9787 (4)	0.8324 (4)	0.56468 (10)	0.0317 (7)	
C32	1.0513 (4)	0.6417 (4)	0.56228 (10)	0.0269 (7)	
H32A	1.0143	0.5796	0.5890	0.032*	
C33	0.9627 (4)	0.5450 (4)	0.52373 (9)	0.0270 (7)	
H33A	0.8260	0.5415	0.5281	0.032*	
C34	1.0350 (4)	0.3543 (4)	0.52206 (10)	0.0285 (7)	
O31	0.8084 (3)	0.8554 (3)	0.57208 (8)	0.0416 (6)	
O32	1.0978 (3)	0.9520 (3)	0.55833 (9)	0.0510 (7)	
O33	1.2503 (3)	0.6346 (3)	0.55866 (8)	0.0381 (6)	
H33	1.279 (5)	0.722 (5)	0.5451 (12)	0.057*	
O34	0.9995 (3)	0.6363 (3)	0.48486 (7)	0.0397 (6)	
H34	1.101 (6)	0.610 (5)	0.4746 (12)	0.060*	
O35	1.1228 (3)	0.2964 (3)	0.49167 (8)	0.0453 (6)	
O36	0.9946 (3)	0.2667 (3)	0.55754 (7)	0.0341 (5)	
H36	1.040 (5)	0.148 (5)	0.5570 (11)	0.051*	
O41	0.5786 (4)	0.5889 (4)	0.60508 (10)	0.0566 (8)	
H41	0.659 (7)	0.681 (6)	0.5942 (14)	0.085*	
H42	0.477 (7)	0.607 (6)	0.5968 (15)	0.085*	

### 4-(4-Fluorophenyl)piperazin-1-ium hydrogen (2R,3R)-tartrate monohydrate (III) Atomic displacement parameters (Å^2^)

**Table:**

	*U*^11^	*U*^22^	*U*^33^	*U*^12^	*U*^13^	*U*^23^
N1	0.0359 (17)	0.061 (2)	0.0389 (18)	−0.0067 (17)	0.0065 (15)	−0.0021 (18)
C2	0.0323 (18)	0.058 (2)	0.049 (2)	−0.0006 (17)	0.0024 (18)	0.004 (2)
C3	0.0353 (19)	0.054 (2)	0.038 (2)	−0.0045 (17)	−0.0052 (17)	−0.0010 (19)
N4	0.0317 (14)	0.0420 (16)	0.0339 (15)	−0.0036 (14)	0.0002 (13)	−0.0030 (14)
C5	0.0322 (17)	0.070 (3)	0.037 (2)	−0.0069 (18)	−0.0011 (17)	−0.004 (2)
C6	0.0374 (19)	0.080 (3)	0.039 (2)	−0.017 (2)	0.0033 (17)	−0.010 (2)
C21	0.0416 (17)	0.0331 (17)	0.0304 (18)	0.0017 (17)	0.0000 (16)	−0.0018 (16)
C22	0.050 (2)	0.041 (2)	0.040 (2)	−0.0004 (18)	−0.0025 (19)	−0.0011 (18)
C23	0.078 (3)	0.056 (2)	0.032 (2)	0.007 (2)	−0.004 (2)	−0.005 (2)
C24	0.069 (3)	0.061 (3)	0.032 (2)	0.010 (2)	0.013 (2)	0.010 (2)
F24	0.101 (2)	0.131 (3)	0.0449 (14)	0.0048 (19)	0.0275 (15)	0.0101 (16)
C25	0.048 (2)	0.066 (3)	0.050 (2)	−0.005 (2)	0.010 (2)	0.010 (2)
C26	0.045 (2)	0.053 (2)	0.040 (2)	−0.0076 (19)	0.0030 (18)	−0.0018 (19)
C31	0.0327 (17)	0.0241 (15)	0.0382 (19)	0.0000 (14)	−0.0036 (15)	0.0015 (14)
C32	0.0241 (14)	0.0230 (14)	0.0337 (18)	−0.0003 (13)	−0.0011 (13)	0.0030 (15)
C33	0.0237 (14)	0.0263 (14)	0.0311 (17)	0.0013 (14)	0.0003 (14)	0.0038 (15)
C34	0.0276 (14)	0.0281 (15)	0.0298 (17)	−0.0001 (15)	−0.0028 (14)	−0.0002 (16)
O31	0.0319 (12)	0.0327 (12)	0.0604 (16)	0.0040 (11)	0.0029 (11)	0.0000 (13)
O32	0.0352 (12)	0.0217 (10)	0.096 (2)	−0.0011 (11)	0.0024 (14)	0.0034 (14)
O33	0.0244 (11)	0.0261 (11)	0.0639 (17)	−0.0006 (10)	−0.0059 (11)	0.0078 (13)
O34	0.0400 (13)	0.0421 (13)	0.0371 (14)	0.0082 (12)	0.0024 (11)	0.0137 (12)
O35	0.0533 (15)	0.0436 (14)	0.0391 (14)	0.0126 (13)	0.0113 (13)	−0.0048 (12)
O36	0.0399 (13)	0.0205 (10)	0.0419 (13)	0.0022 (10)	0.0052 (11)	0.0037 (11)
O41	0.0408 (16)	0.0624 (18)	0.0666 (19)	−0.0045 (14)	−0.0111 (14)	0.0216 (15)

### 4-(4-Fluorophenyl)piperazin-1-ium hydrogen (2R,3R)-tartrate monohydrate (III) Geometric parameters (Å, º)

**Table:**

N1—C2	1.463 (4)	C23—H23	0.9300
N1—C6	1.483 (4)	C24—C25	1.355 (5)
N1—H11	0.87 (4)	C24—F24	1.361 (4)
N1—H12	0.92 (4)	C25—C26	1.384 (5)
C2—C3	1.503 (5)	C25—H25	0.9300
C2—H2A	0.9700	C26—H26	0.9300
C2—H2B	0.9700	C31—O31	1.241 (4)
C3—N4	1.454 (4)	C31—O32	1.248 (4)
C3—H3A	0.9700	C31—C32	1.522 (4)
C3—H3B	0.9700	C32—O33	1.418 (3)
N4—C21	1.414 (4)	C32—C33	1.525 (4)
N4—C5	1.458 (4)	C32—H32A	0.9800
C5—C6	1.503 (5)	C33—O34	1.402 (3)
C5—H5A	0.9700	C33—C34	1.519 (4)
C5—H5B	0.9700	C33—H33A	0.9800
C6—H6A	0.9700	C34—O35	1.204 (4)
C6—H6B	0.9700	C34—O36	1.306 (4)
C21—C22	1.385 (4)	O33—H33	0.80 (4)
C21—C26	1.390 (5)	O34—H34	0.81 (4)
C22—C23	1.387 (5)	O36—H36	0.95 (4)
C22—H22	0.9300	O41—H41	0.95 (5)
C23—C24	1.358 (6)	O41—H42	0.78 (5)
			
C2—N1—C6	111.5 (3)	C21—C22—H22	119.3
C2—N1—H11	115 (2)	C23—C22—H22	119.3
C6—N1—H11	108 (3)	C24—C23—C22	118.4 (4)
C2—N1—H12	108 (2)	C24—C23—H23	120.8
C6—N1—H12	112 (3)	C22—C23—H23	120.8
H11—N1—H12	102 (3)	C25—C24—C23	122.3 (4)
N1—C2—C3	111.5 (3)	C25—C24—F24	118.9 (4)
N1—C2—H2A	109.3	C23—C24—F24	118.8 (4)
C3—C2—H2A	109.3	C24—C25—C26	119.3 (4)
N1—C2—H2B	109.3	C24—C25—H25	120.3
C3—C2—H2B	109.3	C26—C25—H25	120.3
H2A—C2—H2B	108.0	C25—C26—C21	120.6 (3)
N4—C3—C2	110.8 (3)	C25—C26—H26	119.7
N4—C3—H3A	109.5	C21—C26—H26	119.7
C2—C3—H3A	109.5	O31—C31—O32	126.0 (3)
N4—C3—H3B	109.5	O31—C31—C32	118.0 (3)
C2—C3—H3B	109.5	O32—C31—C32	116.0 (3)
H3A—C3—H3B	108.1	O33—C32—C31	112.1 (2)
C21—N4—C3	116.4 (3)	O33—C32—C33	109.4 (2)
C21—N4—C5	115.4 (3)	C31—C32—C33	110.2 (2)
C3—N4—C5	110.2 (3)	O33—C32—H32A	108.4
N4—C5—C6	111.3 (3)	C31—C32—H32A	108.4
N4—C5—H5A	109.4	C33—C32—H32A	108.4
C6—C5—H5A	109.4	O34—C33—C34	111.6 (2)
N4—C5—H5B	109.4	O34—C33—C32	110.7 (2)
C6—C5—H5B	109.4	C34—C33—C32	109.5 (2)
H5A—C5—H5B	108.0	O34—C33—H33A	108.3
N1—C6—C5	109.9 (3)	C34—C33—H33A	108.3
N1—C6—H6A	109.7	C32—C33—H33A	108.3
C5—C6—H6A	109.7	O35—C34—O36	125.5 (3)
N1—C6—H6B	109.7	O35—C34—C33	122.7 (3)
C5—C6—H6B	109.7	O36—C34—C33	111.8 (3)
H6A—C6—H6B	108.2	C32—O33—H33	105 (3)
C22—C21—C26	117.9 (3)	C33—O34—H34	112 (3)
C22—C21—N4	123.3 (3)	C34—O36—H36	113 (2)
C26—C21—N4	118.8 (3)	H41—O41—H42	108 (4)
C21—C22—C23	121.4 (4)		
			
C6—N1—C2—C3	−54.1 (4)	C23—C24—C25—C26	−1.0 (6)
N1—C2—C3—N4	55.9 (4)	F24—C24—C25—C26	−179.4 (3)
C2—C3—N4—C21	168.2 (3)	C24—C25—C26—C21	−0.2 (6)
C2—C3—N4—C5	−57.9 (4)	C22—C21—C26—C25	0.4 (5)
C21—N4—C5—C6	−166.5 (3)	N4—C21—C26—C25	178.2 (3)
C3—N4—C5—C6	59.1 (4)	O31—C31—C32—O33	−173.2 (3)
C2—N1—C6—C5	54.1 (4)	O32—C31—C32—O33	7.7 (4)
N4—C5—C6—N1	−56.8 (4)	O31—C31—C32—C33	64.7 (4)
C3—N4—C21—C22	3.3 (5)	O32—C31—C32—C33	−114.4 (3)
C5—N4—C21—C22	−128.2 (3)	O33—C32—C33—O34	−66.5 (3)
C3—N4—C21—C26	−174.3 (3)	C31—C32—C33—O34	57.2 (3)
C5—N4—C21—C26	54.1 (4)	O33—C32—C33—C34	56.9 (3)
C26—C21—C22—C23	0.6 (5)	C31—C32—C33—C34	−179.4 (2)
N4—C21—C22—C23	−177.1 (3)	O34—C33—C34—O35	4.2 (4)
C21—C22—C23—C24	−1.7 (6)	C32—C33—C34—O35	−118.7 (3)
C22—C23—C24—C25	2.0 (6)	O34—C33—C34—O36	−177.5 (2)
C22—C23—C24—F24	−179.6 (3)	C32—C33—C34—O36	59.5 (3)

### 4-(4-Fluorophenyl)piperazin-1-ium hydrogen (2R,3R)-tartrate monohydrate (III) Hydrogen-bond geometry (Å, º)

*Cg*1 represents the centroid of the ring (C21–C26).

**Table:**

*D*—H···*A*	*D*—H	H···*A*	*D*···*A*	*D*—H···*A*
N1—H11···O36	0.87 (4)	2.31 (4)	2.929 (4)	128 (3)
N1—H11···O35^i^	0.87 (4)	2.17 (4)	2.855 (4)	136 (3)
N1—H12···O41	0.92 (4)	1.83 (4)	2.740 (5)	169 (3)
O33—H33···O32	0.80 (4)	2.19 (4)	2.614 (3)	113 (3)
O33—H33···O34^ii^	0.80 (4)	2.10 (4)	2.805 (3)	146 (3)
O34—H34···O35	0.81 (4)	2.41 (4)	2.702 (3)	102 (3)
O34—H34···O31^ii^	0.81 (4)	2.07 (4)	2.806 (3)	151 (4)
O36—H36···O32^iii^	0.95 (4)	1.53 (4)	2.470 (3)	175 (3)
O41—H41···O31	0.96 (5)	1.82 (5)	2.771 (4)	178 (5)
O41—H42···O33^iv^	0.78 (5)	2.00 (5)	2.754 (4)	163 (5)
C25—H25···*Cg*1^v^	0.93	2.86	3.649 (5)	144

Symmetry codes: (i) *x*−1/2, −*y*+1/2, −*z*+1; (ii) *x*+1/2, −*y*+3/2, −*z*+1; (iii) *x*, *y*−1, *z*; (iv) *x*−1, *y*, *z*; (v) −*x*, *y*−1/2, −*z*+3/2.
